# Circadian activity of *Culicoides oxystoma* (Diptera: Ceratopogonidae), potential vector of bluetongue and African horse sickness viruses in the Niayes area, Senegal

**DOI:** 10.1007/s00436-015-4534-8

**Published:** 2015-05-24

**Authors:** Moussa Fall, Assane G. Fall, Momar T. Seck, Jérémy Bouyer, Maryam Diarra, Thomas Balenghien, Claire Garros, Mame T. Bakhoum, Ousmane Faye, Thierry Baldet, Geoffrey Gimonneau

**Affiliations:** Institut Sénégalais de Recherches Agricoles, Laboratoire National de l’Elevage et de Recherches Vétérinaires, Route du Front de Terre, BP 2057, Dakar, Senegal; Faculté des Sciences et Techniques, Département de Biologie Animale, Université Cheikh Anta Diop, BP 5005, Dakar, Senegal; Centre de Coopération Internationale en Recherche Agronomique pour le Développement, Unité Mixte de Recherche Contrôle des Maladies Animales Exotiques et Emergentes, Campus International de Baillarguet, F34398 Montpellier, France; Institut National de la Recherche Agronomique (INRA), Unité Mixte de Recherche 1309, Montpellier, France

**Keywords:** *Culicoides oxystoma*, Host-seeking activity, Animal-baited trap, African horse sickness, Bluetongue, Senegal

## Abstract

*Culicoides* biting midges (Diptera: Ceratopogonidae) are important vectors of arboviruses in Africa. *Culicoides oxystoma* has been recently recorded in the Niayes region of Senegal (West Africa) and its high abundance on horses suggests a potential implication in the transmission of the African horse sickness virus in this region. This species is also suspected to transmit bluetongue virus to imported breeds of sheep. Little information is available on the biology and ecology of *Culicoides* in Africa. Therefore, understanding the circadian host-seeking activity of this putative vector is of primary importance to assess the risk of the transmission of *Culicoides*-borne pathogens. To achieve this objective, midges were collected using a sheep-baited trap over two consecutive 24-h periods during four seasons in 2012. A total of 441 *Culicoides*, belonging to nine species including 418 (94.8 %) specimens of *C. oxystoma*, were collected. *C. oxystoma* presented a bimodal circadian host-seeking activity at sunrise and sunset in July and was active 3 h after sunrise in April. Daily activity appeared mainly related to time periods. Morning activity increased with the increasing temperature up to about 27 °C and then decreased with the decreasing humidity, suggesting thermal limits for *C. oxystoma* activity. Evening activity increased with the increasing humidity and the decreasing temperature, comprised between 20 and 27 °C according to seasons. Interestingly, males were more abundant in our sampling sessions, with similar activity periods than females, suggesting potential animal host implication in the facilitation of reproduction. Finally, the low number of *C. oxystoma* collected render practical vector-control recommendations difficult to provide and highlight the lack of knowledge on the bio-ecology of this species of veterinary interest.

**Electronic supplementary material:**

The online version of this article (doi:10.1007/s00436-015-4534-8) contains supplementary material, which is available to authorized users.

## Introduction

*Culicoides* biting midges (Diptera: Ceratopogonidae) are small hematophagous insects particularly known for their veterinary importance. Females of several species are implicated in the transmission of viruses, affecting wild and domestic animals worldwide (Mellor et al. 2000). African horse sickness (AHS) is one of the most important and lethal infectious diseases endemic in sub-Saharan Africa affecting equids leading to high mortality rates in susceptible horses (>90 %). Bluetongue (BT) is also an important livestock disease affecting ruminants (Mehlhorn et al. 2007) with a particular concern in sub-Saharan Africa for imported breeds of sheep (Curasson 1925). Although the local sheep breeds in Senegal do not seem to express clinical signs of BT infection, seroprevalence rates remains high among sheep and goats (Lefèvre and Taylor 1983) and the importance of BT for local ruminants remains poorly understand. Moreover, there is little information in West Africa about the biology and ecology of *Culicoides* species, especially those involved in the transmission of AHS and BT viruses. This information is crucial to design and implement vector control strategies, particularly important when safe and efficient vaccines against AHS and BT are not available.

In 2007, an outbreak of AHS virus occurred in Senegal and caused 1169 horse deaths leading to considerable economic losses, estimated to 1.4 million € (Akakpo et al. 2011). Thus, many studies were initiated in the Niayes region of Senegal, particularly affected by the AHS outbreak, to better understand *Culicoides* diversity and their role in the transmission of AHS and BT viruses. These studies identified for the first time *Culicoides oxystoma* Kieffer, 1910 in the Afrotropical region (Bakhoum et al. 2013). This species is abundant in the Niayes area and presents a very aggressive behavior on horses (Diarra et al. 2014; Fall et al. 2015). Although *C. oxystoma* implication in the transmission of AHS and BT viruses in Senegal has never been established (Diarra et al. 2014; Fall et al. 2015), this species is known to be involved in the transmission of bovine arboviruses such as Akabane virus in Japan (Kurogi et al. 1987; Yanase et al. 2005). Furthermore, *C. oxystoma* is a suspected vector of epizootic hemorrhagic disease virus in Israel (Morag et al. 2012) and a potential vector of the bluetongue virus in India (Dadawala et al. 2012). However, there is little information on the biology and ecology of this putative vector, including the description of circadian host-seeking activity, which could have practical implications to allow the development of operational methods for controlling populations of these midges in the field.

Little is known on the host-seeking activity of most Afrotropical *Culicoides* species; on the contrary, some studies have been carried out on Palearctic and Afro-Asiatic species (Mellor et al. 2000). *Culicoides* are mostly crepuscular. For instance, *Culicoides kingi* Austen, 1912 displays two biting peaks: one after sunrise and the other close to sunset (Auriault 1977; El Sinnary et al. 1985; Service 1969). Other species could be active throughout the night, such as reported for *Culicoides venustus* Hoffman, 1925 (Schmidtmann et al. 1980) and *Culicoides distinctipennis* Austen, 1912 (Itoua et al. 1987). Flight activity of midges depends on environmental conditions according to seasons. Indeed, Viennet et al. (2012) highlighted a shift in *Culicoides obsoletus* Meigen, 1818 peak activity from before sunset in spring and autumn to after sunset in summer. These information are crucial to implement vector control strategies such as stabling animals at night to reduce bites by nocturnal vectors (Carpenter et al. 2008). In this context, this study aims to describe the circadian host-seeking activity of *C. oxystoma* according to abiotic conditions observed during four seasons in Senegal.

## Material and methods

### Study area

The entomological survey was conducted at the Laboratoire National de l’Elevage et de Recherches Vétérinaires (LNERV) of the Institut Sénégalais de Recherches Agricoles (ISRA), Hann, Dakar (decimal geographical coordinates: 14.722969, −17.434227). The laboratory is located close to the “Park de Hann,” a forested and swampy area of 0.6 km^2^ inside the urban agglomeration of Dakar, the capital of Senegal. A zoo with approximately 32 different animal species and a horse farm affected by the last epizootic outbreak of AHS in 2007 are located at 200 m and 1 km inside the park, respectively. Rainfall rarely exceeds 500 mm/year with a rainy season ranging from July to October. The dry season (from November to June) can be subdivided into a cold dry season (from November to February) and a warm dry season (from March to June). Ocean vicinity is conducive to a high relative moisture rate ranging from 15 to 90 %, depending on the time of year. The presence and high abundance of *C. oxystoma* in this area have recently been documented (Diarra et al. 2014; Fall et al. 2015).

### Host-baited trap collections

*Culicoides* were collected with a sheep-baited trap (animal weight, 30–35 kg) (Fall et al. 2015). Briefly, the sheep-baited trap was a net box (3.5 × 2.5 × 2.5 m, with mesh of 1.5 × 0.3 mm) with an open space of 15 cm from the ground on each four sides of the trap allowing *Culicoides* to enter in the trap, to engorge or not on the sheep, and avoiding their escape (see Fall et al. 2015 for picture). The sheep was caged in the middle of the trap. The trap was operated during two consecutive periods of 24 h (from 12 p.m. to 12 p.m. the next day) in four different seasons in 2012 and collections were conducted every 3 h (at 12, 15, 18, 21, 0, 3, 6, and 9 o’clock) leading to eight collections per 24-h session. Insects inside the trap were collected with an electric aspirator for 10 to 15 min along the net (not directly on the sheep), which was dropped on the ground during this process. Two collection sessions were carried out during the cold and hot dry season (January and April) and two at the beginning and end of the rainy season (July and October). During experiments, air temperature and relative humidity were recorded every 15 min (thermo-hygrometer HOBO; Onset, Pocasset, MA).

### *Culicoides* identification

After sampling sessions, collection cups were placed at −20 °C for 10 min to kill insects. *Culicoides* were identified under a stereomicroscope (Zeiss, Stemi DV4) using the morphological keys for the Afrotropical region (Boorman 1979; Boorman and Dipeolu 1979; Cornet and Brunhes 1994; Cornet et al. 1974; Glick 1990; Meiswinkel 1989; Meiswinkel 1991). When needed, specimens were dissected and slide-mounted in accordance with the Wirth and Marston technique (1968). *Culicoides* were counted by species and sex, and females were categorized as nulliparous or parous (Dyce 1969), engorged or not, and gravid or not. Samples were stored in Eppendorf tubes with 90° alcohol.

### Statistical analysis

To perform graphical presentation of the results and statistical analysis, the collection results obtained in each of the 3-h duration period were attributed to the median time (i.e., 1:30, 4:30, 7:30, 10:30, 13:30, 16:30, 19:30, 22:30) and associated to average temperature and relative humidity of the same collection periods. Abundance of *C. oxystoma* was modeled using a beta-binomial generalized linear model. We used the temperature, humidity, collection time, and all interactions in the complete model. The best model was selected on the basis of the lowest corrected Akaike information criterion (AICc), and the significance of fixed effects was tested using the likelihood test ratio. We also checked that models fitted the data using a test of goodness-of-fit. All the statistical analyses were conducted using the R software v.3.0.2 (R_Development_Core_Team 2009), with the aods3 package for the beta-binomial generalized linear model and the MuMin package for the implementation of the AICc.

## Results

A total of 441 individuals belonging to nine species were collected (Table 1) over eight 24-h collection sessions (two per season). *C. oxystoma* represented 94.8 % (418 specimens) of the total collections. Males were more abundant than females accounting respectively for 302 (72.2 %) and 116 (27.8 %) individuals. According to season, this higher ratio for males was observed in April (79.7 %) and July (59.8 %) whereas females were more abundant in January (80 %; but only four females were then collected) and October (61.5 %). *C. oxystoma* was collected throughout the four sampling periods. Maximum abundance was observed in April (hot dry season) with 300 individuals collected, and the minimum abundance was in January (cold dry season) with only five individuals caught. For each collection session, approximately half of the caught females were engorged (50, 42.6, and 48.6 % in January, April, and July, respectively) except in October where all females were blood-fed. Gravid females were more abundant in April (47.5 %) and nulliparous females in July (28.6 %).

**Table 1:** Numbers of *Culicoides* collected over two consecutive 24-h periods during four seasons in 2012 using a sheep-baited trap in the Niayes area, Senegal

Species	January						April						July						October					
	Female					Male	Female					Male	Female					Male	Female					Male
	Go	Gr	P	N	Total	Total	Go	Gr	P	N	Total	Total	Go	Gr	P	N	Total	Total	Go	Gr	P	N	Total	Total
*C. oxystoma* (Kieffer, 1910)	2	1	0	1	4	1	26	29	4	2	61	239	17	6	2	10	35	52	16	0	0	0	16	10
*C. distinctipennis* (Austen, 1912)	0	0	0	0	0	0	1	0	0	4	5	1	0	0	0	0	0	0	0	0	0	0	0	1
*C. imicola* (Kieffer, 1913)	0	0	0	0	0	0	0	0	0	1	1	0	0	0	0	0	0	0	1	0	0	1	2	1
*C. austeni* (Carter, 1920)	2	0	0	0	2	0	0	0	0	0	0	0	0	0	0	1	1	0	0	0	0	0	0	0
*C. nivosus* (De Meillon, 1937)	0	0	0	0	0	0	0	0	1	1	2	0	0	0	0	0	0	0	0	0	0	0	0	0
*C. moreli* (Clastrier, 1959)	1	0	0	0	1	0	0	0	0	0	0	0	0	0	0	0	0	0	0	0	0	0	0	1
*C. similis* (Carter, 1921)	0	0	0	0	0	0	0	1	0	0	1	1	0	0	0	0	0	0	0	0	0	0	0	0
*C. leucostictus* (Kieffer, 1911)	0	0	0	0	0	0	0	0	0	0	0	2	0	0	0	0	0	0	0	0	0	0	0	0
*C. murphyi* (Clastrier & Wirth,1961)	0	0	0	0	0	0	0	0	0	0	0	1	0	0	0	0	0	0	0	0	0	0	0	0

*Go* gorged, *Gr* gravid, *P* parous, *N* nulliparous

**Fig. 1:**
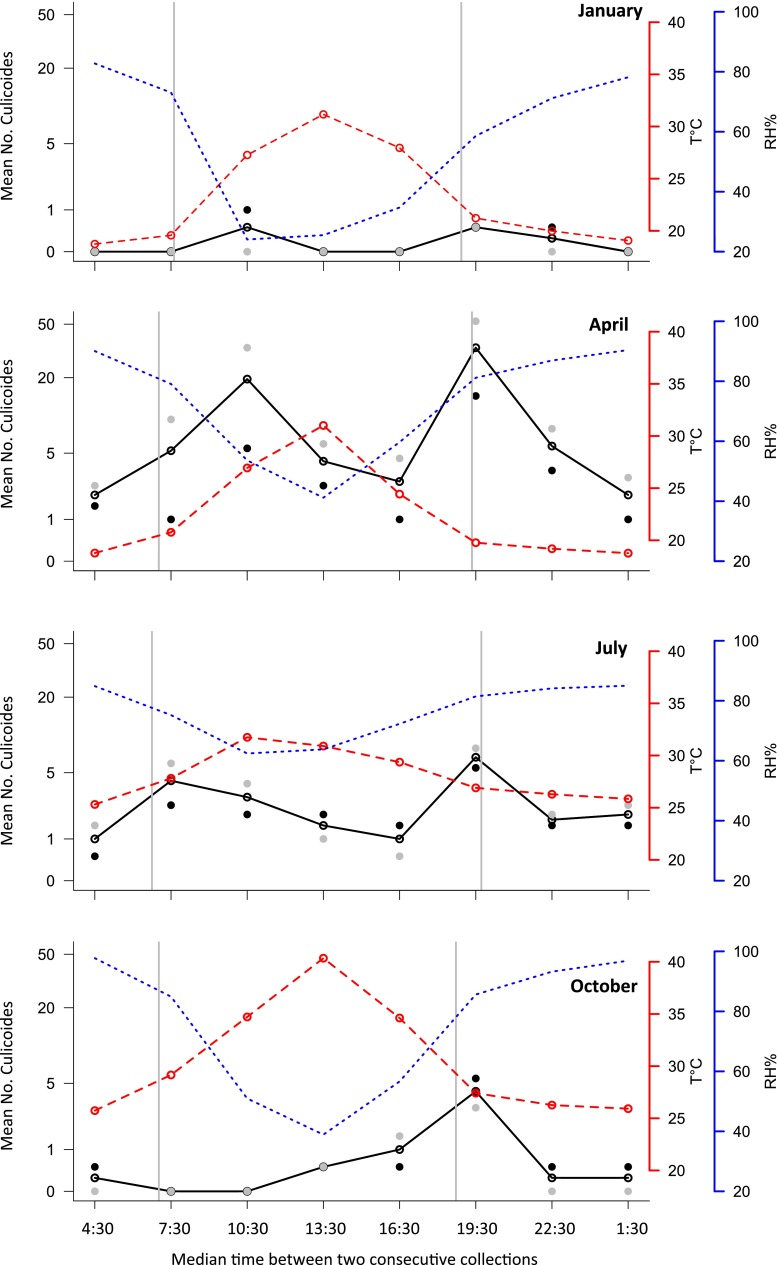
Circadian activity of *Culicoides oxystoma* through four seasons of 2012 using a sheep-baited trap in the Niayes area, Senegal. Legend: *black lines*—the mean number of collected specimens (log 10 scale), *gray dots*—the mean number of males, *black dots*—the mean number of females, *red dashed lines*—average temperatures (°C), *blue dashed lines*—average relative humidity (%). *Gray vertical bars* represent sunrise and sunset time

The circadian host-seeking activity was similar through seasons (Fig. 1), except in October, with a bimodal pattern of host-seeking activity with peaks at sunrise and sunset, although specific differences in amplitude and time were highlighted. Indeed, the time of the activity peaks depended on the season. In April, morning peak activity occurred at least 3 h after sunrise whereas it occurred around sunrise in July and was absent in October. A bimodal activity was also observed in January (Fig. 1), but the number of collected specimens was too low to allow statement about peak activity (Table 1). Evening activity was relatively similar among seasons and took place around sunset.

During the dry season, mean daily temperatures observed in January and April were quite similar: 23.5 °C (17.9–32.6) and 22.8 °C (18.5–33.2), respectively, but humidity rate was higher in April with 71.6 % (38.2–94.6) than in January with 58.4 % (17.4–87.8). Mean temperatures increased thereafter in July: 28 °C (24.8–34.1) with a maximum observed at the end of the rainy season in October with 30.8 °C (24.4–44.2). Despite high variations of relative humidity recorded during trap collections in July and October (57.5–94.1 and 28.7–98.8 %, respectively), the average values of the daily humidity were similar in both collection periods (see additional table 1 for range value per collection session). Although differences in temperatures were observed during the four sampling periods, morning peaks activities always occurs around 27 °C whereas it varied between 20 °C (April) and 27 °C (July and October) at the sunset according to seasons. However, in January, collections were positive in the morning at temperature around 27 °C and after the sunset around 20 °C, but in both cases very few individuals were collected (Table 1). Humidity was variable according to season, but decreasing the morning and increasing the afternoon.

The numbers of male and female collected over the year were strongly correlated (Pearson’s correlation test, *r* = 0.897, *P* < 0.001) indicating a similar pattern of activity (Fig. 1). According to this result, male and female abundance data were pooled in the model in order to gain power. The highest activity was observed at sunrise and sunset (Fig. 1) with significant effects of temperature (*Z* value = 3.685, *P* < 0.001) and humidity (*Z* value = −2.223, *P* = 0.026). Overall, morning activity was related to temperature increase and humidity decreases whereas the reverse was observe in the evening. However, the time period was much more important than these two effects, with the lowest activity observed between 15 and 18 h (median of 16:30, with a significantly lower activity than all other time periods; Fig. 1).

## Discussion

This study present the first data on circadian activity of *C. oxystoma* during different seasons, a newly recorded species for Senegal (Bakhoum et al. 2013) and a putative vector of AHS and BT viruses in the Niayes region of Senegal (Diarra et al. 2014; Fall et al. 2015). Although this species was collected throughout the four sampling periods, maximum abundances were observed in hot dry season and at the beginning of the rainy season. Circadian bimodal activity at an animal host was observed around sunset and sunrise with a similar activity pattern between males and females and abiotic parameters, temperature, and humidity having significant effects on midge activity.

The bimodal activity observed for *C. oxystoma* (around sunset and sunrise) has been described for *Culicoides* species in the Afrotropical region (Auriault 1977; El Sinnary et al. 1985) and the lower activity during daylight has been attributed to abiotic parameters. Flight activity is influenced by meteorological parameters such as variation in light intensity, temperature, and humidity, which are closely correlated (Auriault 1977; El Sinnary et al. 1985; Tsutsui et al. 2011; Zimmer 2007). Indeed, Blackwell (1997) highlighted that *Culicoides impunctatus* Goetghebuer, 1920 activity is positively correlated to both relative humidity and rainfall and presents negative relationships with wind velocity. Temperatures above and below certain limits restrict *Culicoides* activity (Auriault 1977; Zimmer et al. 2008) and the 3-h shift in morning activity of *C. oxystoma* in July is probably due to the high increase in temperature during this season. Indeed, morning collections in January, as well as morning peak activity in April and July occurred around 27 °C. Beyond this temperature, flight activity decreases, suggesting thermal limits for *C. oxystoma* activity. In the same time, humidity was always decreasing during these morning activity periods. The absence of morning peak activity in October was probably due to high temperature (the hottest month of the year) early in the morning but also other parameters like wind speed (not recorded in this study), which could disrupt the odor flows emanating from the host or reduce flight activity (Walker 1977). Although variable between seasons in amplitude, flight activity was always more pronounced at sunset as for other *Culicoides* species in the Afrotropical region and seemed always limited between 20 and 27 °C and related to a humidity rate of over 80 % (Auriault 1977; El Sinnary et al. 1985; Walker 1977).

The close association found between male and female *C. oxystoma* activity is intriguing. To our knowledge, this is the first time that males are so abundantly collected using an animal-baited trap. Rawlings et al. (1998) captured more males than females at dry season using light trap in Gambia. However, this result is unusual and males are rarely caught in light trap collections. The relative high abundance of males may be the result of trap position, set up between trees to avoid direct animal exposure to the sun. Indeed, it has been highlighted that *Culicoides* are generally found during daytime in shaded vegetated areas and/or the underside of leaves (Zimmer 2007). Moreover, males are nectar and pollen feeder and rest preferentially in shrubs or trees (Zimmer et al. 2008). It could also be suggested that male abundance is in relation to the ongoing reproductive status of the population (i.e., emergence of a new generation). Indeed, as in many dipterans, the abundance of males in certain periods indicates the ongoing emergence of a new generation of adults (Downes 1969), as males usually emerge a little bit earlier than females, being ready for mating as soon as females emerge. However, few nulliparous females were collected for each collection session and approximately half of the caught females were engorged preventing reproductive status determination. Another hypothesis could be related to *C. oxystoma* mating behavior. There is not much information on *Culicoides* mating behavior (Campbell and Kettle 1979; Gerry and Mullens 1998; Zimmer et al. 2008). Campbell and Kettle (1979) described *Culicoides brevitarsis* Kieffer, 1917 mating behavior indicating that males and females swarms and mates around ecological markers such as shadows cast by clumps of living grass slightly elevated or near to cattle (<10 m). Observations of mating behavior near and on a host calf were made at a nearby dairy for *Culicoides sonorensis* Wirth & Jones, 1957 (Gerry and Mullens 1998). Males swarmed 1–2 m downwind of a restrained calf and 0.3–1.0 m above ground level. Males were also observed coupled with blood-feeding females on the calf venter (especially umbilicus and teats). Swarming reproductive behavior has been well described for many Diptera species (Downes 1969). Mating occurs in and around swarms of numerous males that form at specific sites at sunset and last for about 20 min. Females typically approach a swarm, acquire a mate, and leave in copula. This typical swarming reproductive behavior could explain the high abundance of males collected in our study. Although no specific studies have been conducted on *C. oxystoma* mating behavior, our results suggest that the animal host could also be a “swarm marker” in the reproduction of this species according to its shape and/or odor litter. Because our sampling collections were not directly conducted on the host, it could not be excluded that *C. oxystoma* mate on the host (Gerry and Mullens 1998). Further studies are needed to better understand the reproductive behavior of this species.

Overall, the total number of *Culicoides* collected during present study seems lower than previous studies performed in the same area (Diarra et al. 2014; Fall et al. 2015). In our study, a sheep-baited trap was preferred to an UV-light/suction trap because the latter misestimate the biting rate and/or attack rate on a host animal (Viennet et al. 2011). Moreover, a specific shortcoming of the UV-light/suction trap is that it is able only to monitor *Culicoides* that are active nocturnally (being unable to attract and collect species which are active diurnally). Diurnal livestock attack rates need to be quantified as they have implications for the transmission of BT and AHS viruses. There is no information on *C. oxystoma* host preference, and we could suggest that sheep is not the preferential host of this putative vector species explaining why relatively few individuals were collected. Moreover, other potential hosts present in the vicinity such as mammals inside the zoo (200 m) and horses (1 km) may have contributed to the low collection rate of *C. oxystoma* but also other *Culicoides* species. Overall, our result highlights that *C. oxystoma* host-seeking and blood-feeding activity mainly occurred at sunrise and sunset and to a lesser extent during the day. This behavioral activity has implication in the protection of valuable animals in Senegal such as horse and imported breeds of sheep. Indeed, in order to reduce host/vector contacts, animals could be sheltered before sunset and release after sunrise as it has been proposed to protect horses from AHS vectors such as *Culicoides imicola* Kieffer, 1913 in South Africa (Meiswinkel et al. 2000). However, the effectiveness of this measure to limit their risk to be bitten by *C. oxystoma* and then to be infected by *Culicoides*-borne pathogens is highly dependent in the degree of endophagy and exophagy of this species. However, there is no information on *C. oxystoma* endo-/exophagy behavior which renders practical vector-control recommendations difficult to provide.

This study represents the first data on *C. oxystoma* circadian activity, which demonstrated a bimodal activity at sunset and sunrise as described for other species. Although *C. oxystoma* exhibits mainly a crepuscular activity, its ability to be active after the sunrise in optimal temperature and humidity conditions makes us question the possibility of a daytime transmission of BT virus to sheep and AHS virus to horses. These results show that we should not only be limited to develop tools and methods to protect the animals at night but also during the day, especially at sunrise and sunset. The high presence of males in collections, the strong correlation of their daily activity pattern to females, and the overall low number of specimens collected highlight the lack of knowledge on the bio-ecology (i.e., mating and host preference behaviors) of this important species. Further research is needed to gain a better understanding of the bio-ecology of this species and its role as potential vector of AHS and BT viruses in Senegal.

## Electronic supplementary material

Additional table 1 Results of adults *Culicoides oxystoma* (males and females) collected during 2 days for 4 seasons and minimal and maximal data for temperature and relative humidity (PDF 28 kb)
