# Supplementary material for: Circadian activity of Culicoides oxystoma (Diptera: Ceratopogonidae), potential vector of bluetongue and African horse sickness viruses in the Niayes area, Senegal
Source: Parasitol Res. 2015 May 24;114(8):3151–8. doi: 10.1007/s00436-015-4534-8 (PMC4513201; doi:10.1007/s00436-015-4534-8)
Supplement: Supplementary file 1 — Results of adults Culicoides oxystoma (males and females) collected during 2 days for 4 seasons and minimal and maximal data for temperature and relative humidity (PDF 28 kb) [file 436_2015_4534_MOESM1_ESM.pdf]

Season	January		April		July		October	
Capture session	1	2	1	2	1	2	1	2
C. oxystoma (n)	5	0	39	261	76	11	2	24
Temperature (°C) [min-max]	[17.6-32.6]	[19.4-32.5]	[18.5-31.2]	[28.5-33.2]	[24.8-30.8]	[25.1-34.1]	[25.9-42.9]	[24.4-44.2]
Relative Humidity (%) [min-max]	[17.4-84.8]	[30.6-87.8]	[39.3-89.7]	[38.2-94.6]	[59.0-79.9]	[57.5-94.1]	[36.9-98.8]	[28.7-97.5]
